# Methane emissions in triple rice cropping: patterns and a method for reduction

**DOI:** 10.12688/f1000research.20046.6

**Published:** 2021-02-15

**Authors:** Masato Oda, Huu Chiem Nguyen

**Affiliations:** 1 Japan International Research Center for Agricultural Sciences, Tsukuba, Japan; 2 Department of Environmental Science, Can Tho University, Can Tho, Vietnam

**Keywords:** Greenhouse gases, Mekong Delta, Methanogenesis inhibition, Rice straw, Flooding, Methane reduction

## Abstract

The Mekong Delta paddies are known as hotspots of methane emission, but these emissions are not well studied. We analyzed methane emission patterns based on monitoring data from typical triple rice cropping paddies collected over 5 years. We found that the total emissions in a crop season doubled in the second crop, tripled in the third crop, and reset after the annual natural flood of the Mekong River. The emission peaks occurred around 0 to 3 weeks after starting irrigation, then gradually decreased. In general, the main source of emitted methane is rice-derived carbon by current-season photosynthates and the emission peaks at the rice heading stage. However, the contribution of the rice-derived carbon is negligible in the hotspot paddies because total emission is high. The increase in emission levels from the first to the third crop can be explained by the accumulation of rice residue from the preceding crops, especially rice straw incorporated into the soil. The reset of emission levels after the annual flood means that the rice straw is decomposed without methanogenesis in water with dissolved oxygen. Thus, the annual emission pattern shows that avoiding rice straw incorporating into soil and decomposing rice straw in paddy surface-water reduces methane emissions.

## Introduction

Vietnam is the world’s fifth largest rice producer ( FAO 2018). The Mekong Delta produces the half (23.8 million tons) ( General Statistics Office of Vietnam 2016). The climate of tropical monsoon (Am) enables high productivity by triple rice cropping (cropping three times a year). Rice paddies are a methane emission source, and the Mekong Delta is a hotspot ( Arai *et al.*, 2018; Werner *et al.*, 2016). The high emissions are caused by the rice straw incorporation ( Oda & Chiem, 2019). However, the methane emission of triple rice cropping has not been well studied ( Vo *et al.*, 2018).

The Mekong’s natural flood of two months (starting from around late September to late October) limits the rice cultivation period. The 1 ^st^ crop (winter-spring) begins after the natural flood, then after harvesting the rice straw is incorporated into the soil. The 2 ^nd^ (spring-summer) and the 3 ^rd^ crop (summer-autumn) follows without interval. Just after the 3 ^rd^ crop, the natural flood starts so the straw is left on the paddies and decomposes under the floodwater. Then, the 1 ^st^ crop begins again without incorporation of the straw in the soil (field leveling only), because they are sufficiently decomposed by that time.

Can Tho University (CTU) and the Japan International Research Center for Agricultural Sciences (JIRCAS) conducted joint research and monitored methane emissions in typical triple rice cropping paddies for 5 years (for a total of 15 crops). This paper is a specific analysis of a part of the data set from this project. We aimed to clarify the pattern of the methane emission.

## Methods

### Site description

The observation was conducted on a farmer’s paddies (three fields) managed by the above typical triple-cropping in Thuan Hung village (10°22' N, 105°58' E), Thot Not district, Can Tho city, Vietnam from 2011 to 2016. The soil is alluvium soil (Aquic Tropaquepts; 52% clay, 48% silt, <1% sand). Normally, from May to October is the rainy season. The farmer managed the water with continuous flooding. The low dike system could not protect their paddy fields from the annual flood. The rice ( *Oryza sativa*) variety Jasmine was used for the 1 ^st^ crop, and OM501 was used for the 2 ^nd^ and 3 ^rd^ crop every year. The average number of growth days per crop were 103, 89, and 92, for the 1 ^st^, 2 ^nd^, and 3 ^rd^ crops, respectively. The average intervals between the 1 ^st^ and the 2 ^nd^ crop and the 2 ^nd^ and the 3 ^rd^ crop were 5.6 and 6.6 days, respectively. The average rice straw dry weight per crop were 9.0, 9.3, and 7.4 (Mg ha ^–1^), for the 1 ^st^, 2 ^nd^, and 3 ^rd^ crops, respectively, and the whole amount were returned. Rice straws were incorporated into the soil after the 1 ^st^ and 2 ^nd^ crop but left on the ground after the 3 ^rd^ crop. Note, we confirmed that no rice straw (the source of methanogenesis) was lost to the floodwater. This study was conducted with the approval of the farmer.

### Methane measurement

We used the closed chamber method established by NARO and IRRI ( http://globalresearchalliance.org/research/paddy-rice/), and the measurements were taken at 8 a.m. (ca. 90% of the average daily emissions). In periods of natural flood, chambers with attached Styrofoam floats were used. Measurements were taken once a week throughout the rice growing stage, but every 3 days for 2 weeks after seeding, heading stage, and around draining ( Oda & Chiem, 2019).

### Statistical analysis

The cumulative CH4 emissions were calculated by linear interpolation. Descriptive statistics were calculated using Microsoft Excel 2016.

## Results

### Emission level

**Figure 1.**
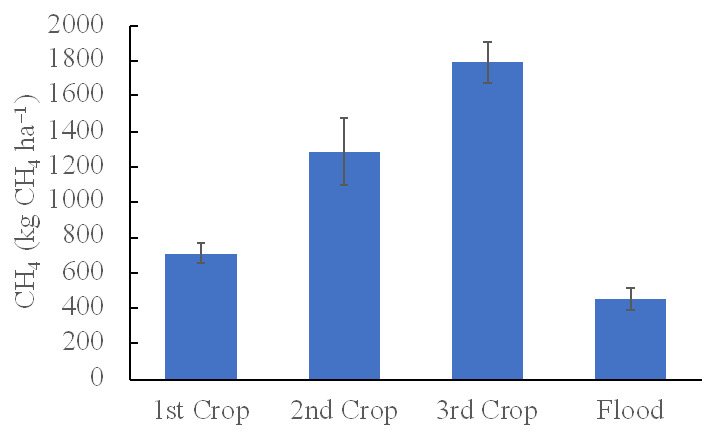
Total CH _4_ emissions. Five-year average of CH _4_ emissions of triple-cropped rice paddies in the Mekong Delta (2011–2016). Bars represent 95% CI (n = 5).

According to the IPCC guidelines, standard methane emissions over 100 days of continuously flooding rice cropping are 130 kg ha ^−1^ crop ^−1^. Wassmann *et al.* (1996) reported very high emissions (160–240 kg ha ^−1^ crop ^−1^) from double cropping rice paddies in the Philippines after organic matter incorporation. However, we observed larger emissions (710, 1290, and 1789 kg ha ^−1^ crop ^−1^), for the 1 ^st^, 2 ^nd^, and 3 ^rd^ crops in average, respectively. Vo *et al*. (2018) measured the same level of emission in the Mekong delta (ca. 900 kg CH4 ha ^–1^ crop ^–1^). The emission level doubled in the 2 ^nd^ crop, and tripled in the 3 ^rd^ crop, then reset after the natural flood ( Figure 1). Furthermore, the total emissions during the flood period and the 1 ^st^ crop was lower than that of the 3 ^rd^ crop ( Figure 1).

### Emission pattern in each cropping

**Figure 2.**
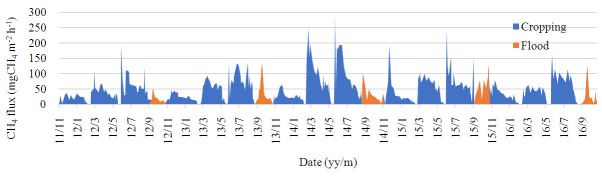
Actual CH _4_ emissions. CH _4_ emissions of triple crop rice paddies in the Mekong Delta (2011–2016). Data are the mean of three replications. Irrigation started 6 days after seeding and drained about 10 days before harvesting. The average days of interval between the harvesting and seeding was 6.1 days. The heading stage of the rice is about a month before drainage.

Oda & Chiem (2019) indicated three types of methane emission patterns during the rice growth period. Generally, the emissions peak at the heading stage due to the methanogenesis substrate provided by the present rice. Another pattern can occur with an additional peak at the early stage of rice growth if organic matter was incorporated beforehand. The third is the pattern in the triple rice cropping. The emission peaks at the early stage of rice growth, then gradually decreases; the peak at the heading stage is undetectable because of the high emission levels. This means the contribution of the rice-derived carbon is small. The pattern of methane emission in each crop season was the same as the study of Oda & Chiem (2019). The emissions began with irrigation, reached peaks from 0 to 3 weeks after the start of irrigation (see *Extended data*, Supplemental figure; Oda, 2019b), and gradually decreased, and the peak at the heading stage was undetected. Furthermore, the emissions during the natural flood appeared to be a continuation of the emissions of the 3 ^rd^ crop ( Figure 2).

## Discussion

### Increase of emission

The total emissions in a crop season doubled in the second crop, tripled in the third crop. This can be explained by the accumulation of rice residue from the preceding crops, especially by the rice straw incorporated into the soil, because the amount of the present rice-derived carbon at emission peak (small plant just after sowing) is small ( Oda & Chiem, 2019). Incorporation of organic matter just before rice cultivation largely increases the methane emission in the paddy field ( Wassmann *et al.*, 1996). 

### Reset of emission

In contrast, that just after the 3rd crop, the natural flood starts so the straw is left on the paddies. No rice straw is incorporated into the soil before the flood period. That results in the reduction of CH _4_ emission. The reset of emission levels after the annual flood means that the rice straw is decomposed without methanogenesis in water because the water includes dissolved oxygen. Convection of surface water transports new water to rice straws and new oxygen replenishes from the atmosphere when reducing the concentration of dissolved oxygen. Thus, the redox potential of water hardly achieves the level of methane generation. In fact, the rice straw on the paddy surface contribute to little methane emission because the emissions during the natural flood appeared to be a continuation of the emissions of the 3 ^rd^ crop. If the rice plant residues were incorporated into the soil, the total emission of the flood period should be higher than that of the 3 ^rd^ crop. Because the accumulation of organic matter is larger. In addition, although the absence of rice-derived carbon, the absence of rice plants doubles the methane emission from the field because of the lack of methanogenesis inhibition by rice plants ( Oda & Chiem, 2019). A portion of emission in the first crop will be caused by incorporation of the remaining rice straw related to the leveling of the field.

### Method for methane reduction

Our results indicate that the main cause of the increase in methane emissions was the incorporation of rice straw into the soil. In contrast, decomposing rice straw in paddy surface-water generated less methane. Thus, decomposing rice straw in paddy surface-water is an effective method to reduce methane emissions in this area. In developing a practical technologies, environmental sustainability or socioeconomic considerations must be considered.

## Conclusion

We analyzed the methane emission patterns of triple rice cropping paddies in the Mekong Delta. Methane emissions increased with rice straw incorporation into the soil. The natural flood resulted in decomposition occurring in the water, leading to less methane emission. Therefore, the annual emission pattern suggests that decomposing rice straw in paddy surface-water is an effective method to reduce methane emissions. In developing a practical technologies, environmental sustainability or socioeconomic considerations must be considered. The development of practical technology to attain this reduction is a subject for a future study.

## Data availability

### Underlying data

Figshare: Methane emission from triple cropping rice field. https://doi.org/10.6084/m9.figshare.9757934.v1 ( Oda, 2019a).

### Extended data

Figshare: Methane flux of days after transplanting. https://doi.org/10.6084/m9.figshare.9746006.v1 ( Oda, 2019b).

Data are available under the terms of the Creative Commons Attribution 4.0 International license (CC-BY 4.0).
