# Supplementary material for: Methane emissions in triple rice cropping: patterns and a method for reduction
Source: F1000Res. 2021 Feb 15;8:1675. Originally published 2019 Sep 23. [Version 6] doi: 10.12688/f1000research.20046.6 (PMC7059783; doi:10.12688/f1000research.20046.6)
Supplement: Peer review history

# Peer review: Reviewer response for version 6

**DOI:** 10.5256/f1000research.29139.r79578

**Published:** 2021-03-15

**Authors:** Arika Bridhikitti

**Affiliations:** 1 Environmental Engineering and Disaster Management Program, Mahidol University Kanchanaburi Campus, Kanchanaburi, Thailand

Though having 6 revisions, this manuscript is still difficult to understand. The author delivered the simple thing in a difficult way. Repeated same issues many times but obscured some issues. My previous suggestions are not fully acknowledged in this manuscript and this time may not as well.

 Here are the difficulties:

1. Abstract:
  In general, the main source of emitted methane is rice-derived carbon by current-season photosynthates and the emission peaks at the rice heading stage. However, the contribution of the rice-derived carbon is negligible in the hotspot paddies because total emission is high”
  The above sentences come from nowhere. Photosynthesis is not included in this study and CH4 should not come from photosynthesis. The second sentence is not reasonable since the carbon is from the decomposition of rice straw.
2. Emission pattern in each cropping:
  Three types of methane emission patterns are not clear. When is the heading stage? When is the early stage of rice growth? What is the methanogenesis substrate from the present rice? The authors also mention that no peak at the heading stage because of the high emission levels. What mean by this sentence? Is it higher than the detection limit of the device? Overall, please rewrite this section.
3. Figure 2:
  Please make notices on the seeding, harvesting, heading, flooding stages in the figure.

Is the work clearly and accurately presented and does it cite the current literature?

Yes

If applicable, is the statistical analysis and its interpretation appropriate?

Not applicable

Are all the source data underlying the results available to ensure full reproducibility?

No

Is the study design appropriate and is the work technically sound?

Yes

Are the conclusions drawn adequately supported by the results?

Partly

Are sufficient details of methods and analysis provided to allow replication by others?

No

Reviewer Expertise:

Environmental Engineering

I confirm that I have read this submission and believe that I have an appropriate level of expertise to confirm that it is of an acceptable scientific standard, however I have significant reservations, as outlined above.

# Peer review: Reviewer response for version 6

**DOI:** 10.5256/f1000research.29139.r79579

**Published:** 2021-03-15

**Authors:** Kazuyuki Yagi

**Affiliations:** 1 The Joint Graduate School of Energy and Environment (JGSEE), Center of Excellence on Energy Technology and Environment, King Mongkut’s University of Technology Thonburi, Bangkok, Thailand

I read the revised manuscript (ver. 6) and the Author Response (dated on 15 Feb 2021) to my comments on its ver. 5 (13 Jul 2020) that was reported to me on 15 Feb 2021. As a result, I recognize that some improvements were added to the revised manuscript. However, the latest version of the manuscript (ver. 6) still has a number of problems in the presentation and interpretation of the experiment and its results.

 Judging from the ‘Author Response (dated on 15 Feb 2021)’, I am afraid that the authors make a big misunderstanding about my comments that were repeatedly asked in Reviewer Reports. The authors insisted that “You didn't give us any response so we understood that you accepted our response”. However, this is wholly to misconceive my comments to the previous version (ver. 5) on 13 Jul 2020. In this previous response, I have requested to respond my previous comments to ver. 2 and 4. However, many of these comments are not appropriately responded to in the latest version.

 Consequently, I have no other choice than reporting that the response of the authors is still unsatisfactory to accept. I am listing problems of the manuscript below and request authors to revise the manuscript in response to each comment. I hope the authors also explain how they responded to each of the comments, as a general procedure in responding to the peer review in most scientific journals. I fully agree with your suggestion that we should reduce the effort of the Editorial Team. But if you think so, you should respond to the comments from reviewers completely and conscientiously.

**Fundamental problems:**

1. Introduction: Scientific questions and objectives of this study are not clear. Background (Why did you study methane emissions from paddy fields? What gaps of knowledge did you try to approach?) and objectives of the study should be clearly presented. It seems that this study has aimed at more than that mentioned in the last sentence of ‘Introduction’, not only to clarify the pattern of the methane emissions but also to propose a possible option for reducing the emissions.
2. There is a fundamental lack of discussion with referring to previous studies by other researchers who reported methane emissions from paddy fields. There are a number of reports on the intensity, patterns, and reduction options for methane emissions from paddy fields. A sweeping revision is necessary in the Discussion section.

**Other specific problems:**

1. Abstract, line 2: The statement that ‘these emissions are not well studied’ is incorrect. A number of field measurements for methane emissions from paddy fields in the Mekong Delta have been published, such as Vo et al., 2018 (already cited in this paper), and references cited in this paper.
2. Abstract, line 11: It is not reasonable to state that the contribution of the rice-derived carbon is ‘negligible’. It is stated to be ‘small’ in the text.
3. Introduction, 1st paragraph, line 2: It is necessary to clarify ‘the half’ of what.
4. Introduction, 2nd paragraph, last phrase: It is necessary to indicate the evidence or possible mechanisms that the straw in the soil is sufficiently decomposed before the beginning of the 1st crop.
5. Methods, Site description: Chemical properties of the soil at the site are suggested to report, in addition to the type and texture, because the information is essential for discussing the intensity of methane emissions from paddy fields. I would suggest reporting, at least, organic carbon content and pH.
6. Methods, Methane measurement: How many replications of the measurement did you conduct? It is necessary to mention that the data for emission rates from each replication are averaged.
7. Methods, Statistical analysis: It is necessary to explain ‘descriptive statistics’ more, including the method to test statistical significance.
8. Results, Emission level, lines 6–8: The reasons for larger emissions of CH4 in the study field should be discussed here or in ‘Discussion’.
9. Results, Emission level, lines 10–12: The emission level doubled in the 2nd crop, and tripled in the 3rd crop, “ compared with that in the 1st crop,” then … (the phrase in “ “ should be added). Same corrections should be made at Abstract, lines 4–7 and Discussion, Emission pattern, lines 1–3.
10. Results, Emission pattern in each cropping, lines 1–11: This statement is not the results in this study. Therefore, it should be moved to Discussion.
11. Results, Emission pattern in each cropping, lines 11–12: It is questionable to discuss the emission patterns with reference to the study of Oda & Chiem (2019) because it reported only the results in the winter-spring season. A wider discussion with referring other studies is expected.
12. Discussion, Reset of emission lines 17–20: The discussion about the effect of rice plants on the methane emissions is just a speculation from the result of Oda & Chiem (2019). Therefore, it cannot be concluded.
13. Discussion, Method for methane reduction, the last sentence: The statement is vague. It is requested to specify how practical technologies are considered by deducing from this study. Also, the same comment to Conclusion lines 7–9.
14. Figure 1: What do the error bars indicate? Are they 95% CI of inter-annual variations of emissions? It is necessary to clarify it by adding in the caption.
15. Figures 1 and 2: The period of data ‘Flood’ is confusing. It is recommended to correct it with ‘Annual flood’.
16. Many inappropriate usages of English grammar and expressions are found throughout the manuscript. Therefore, it is recommended to edit the manuscript by an English expert.

Is the work clearly and accurately presented and does it cite the current literature?

No

If applicable, is the statistical analysis and its interpretation appropriate?

No

Are all the source data underlying the results available to ensure full reproducibility?

Partly

Is the study design appropriate and is the work technically sound?

Yes

Are the conclusions drawn adequately supported by the results?

Partly

Are sufficient details of methods and analysis provided to allow replication by others?

No

Reviewer Expertise:

biogeochemistry, soil science

I confirm that I have read this submission and believe that I have an appropriate level of expertise to state that I do not consider it to be of an acceptable scientific standard, for reasons outlined above.

# Peer review: Reviewer response for version 5

**DOI:** 10.5256/f1000research.27595.r65618

**Published:** 2020-07-13

**Authors:** Kazuyuki Yagi

**Affiliations:** 1 The Joint Graduate School of Energy and Environment (JGSEE), Center of Excellence on Energy Technology and Environment, King Mongkut’s University of Technology Thonburi, Bangkok, Thailand

I read the revised manuscript (ver. 5) and the Author Response (dated on 23 Mar 2020) to my comments on its ver. 4 (12 Mar 2020) that was reported me on 26 Jun 2020. Although some revisions have made in the manuscript, the latest version of the manuscript (ver. 5) still has a number of problems and misunderstandings in presentation and interpretation of the experiment and its results. I do not recognize the revisions in response to most of my previous comments to ver. 2 and 4. As a result, in conclusion, I have to report that the response is still unsatisfactory to accept.

Is the work clearly and accurately presented and does it cite the current literature?

No

If applicable, is the statistical analysis and its interpretation appropriate?

No

Are all the source data underlying the results available to ensure full reproducibility?

Partly

Is the study design appropriate and is the work technically sound?

Yes

Are the conclusions drawn adequately supported by the results?

Partly

Are sufficient details of methods and analysis provided to allow replication by others?

No

Reviewer Expertise:

biogeochemistry, soil science

I confirm that I have read this submission and believe that I have an appropriate level of expertise to state that I do not consider it to be of an acceptable scientific standard, for reasons outlined above.

# Response

**Published:** 2020-07-14

**Authors:** Masato Oda

**Affiliations:** Japan International Research Center for Agricultural Sciences (JIRCAS), Japan

We hope the misunderstandings should be clarified in the response so that we could know the misunderstandings. You didn't give us any response so we understood that you accepted our response. You and we should reduce the effort of the Editorial Team.

# Peer review: Reviewer response for version 5

**DOI:** 10.5256/f1000research.27595.r65620

**Published:** 2020-06-29

**Authors:** Arika Bridhikitti

**Affiliations:** 1 Environmental Engineering and Disaster Management Program, Mahidol University Kanchanaburi Campus, Kanchanaburi, Thailand

In summary, the manuscript is still lacking in scientific sound and requires extensive revision. The contradictions between this finding and the previous studies have not yet been logically justified. Oda and Chiem (2019) mentioned the methanogenesis inhibited by rice plants, but this study shows higher methane during the cropping cycles than those in the non-cropping phase. The authors said that "No rice straw is incorporated before the flood period. That is the difference in the conclusion of Oda and Chiem (2019)."  It is not clear to me what "incorporated" means and how it results in the different outcomes between these two studies. Is It is sound inconsistency with the statement that "We confirmed that no rice straw (the source of methanogenesis) was lost to the floodwater."

 The authors persuaded that the no-plant flood mentioned in Oda and Chiem (2019) had no rice straw, but in this study, there was straw left in the field during the natural flood. The authors also conclude that soil organic alone under flooding promotes methane emission, but soil organic with rice straw incorporated was not. The authors said that the reason is due to sufficient DO. This conclusion was puzzling. How to prove that the flood in the previous study has insufficient DO, but there was adequate for this study? The authors, therefore, should discuss more the pathways of C losses (CH _4_, CO _2_, SOC, Dissolve forms, bio uptake, etc.) from the paddy field during the natural flood.

 The authors point out that decomposing rice straw in non-cropping flooding water generates less methane under sufficient DO so that they recommend the audiences to experiment as a way to minimize methane emission. This recommendation might not be useful and need to open for multidisciplinary discussions. First, the cropping environment factor should be discussed in response to the limitation and possible adoption of this CH _4_ mitigation practices in other areas. Second, socioeconomics should be firmly pointed out since minimizing cropping cycles may not be socially acceptable. Thrid, environmental sustainability by CH _4_ mitigation may not correct since the conversion of CH _4_ to other carbon forms could also result in additional ecological problems.

Is the work clearly and accurately presented and does it cite the current literature?

Yes

If applicable, is the statistical analysis and its interpretation appropriate?

Not applicable

Are all the source data underlying the results available to ensure full reproducibility?

No

Is the study design appropriate and is the work technically sound?

Yes

Are the conclusions drawn adequately supported by the results?

Partly

Are sufficient details of methods and analysis provided to allow replication by others?

No

Reviewer Expertise:

Environmental Engineering

I confirm that I have read this submission and believe that I have an appropriate level of expertise to state that I do not consider it to be of an acceptable scientific standard, for reasons outlined above.

# Response

**Published:** 2020-07-14

**Authors:** Masato Oda

**Affiliations:** Japan International Research Center for Agricultural Sciences (JIRCAS), Japan

"Incorporation" means rice straw is incorporated into the paddy soil by tillage. We ask you to read the references.

# Response

**Published:** 2020-08-26

**Authors:** Masato Oda

**Affiliations:** Japan International Research Center for Agricultural Sciences (JIRCAS), Japan

1) It is not clear to me what "incorporated" means and how it results in the different outcomes between these two studies.

We've noticed that a sentence is unclear to indicate the work of Oda & Chiem *2019).

We revised the manuscript as follows.

Discussion- Emission pattern

"The total emissions in a crop season doubled in the second crop, tripled in the third crop, and reset after the natural flood. This can be explained by the accumulation of rice residue from the preceding crops, especially by the rice straw incorporated into the soil **.** Because the contribution of the present rice-derived carbon is small ( Oda & Chiem, 2019). **Incorporation of organic matter just before rice cultivation largely increases the methane emission in the paddy field ( Wassmann et al., 1996). However, **that just after the 3rd crop, the natural flood starts so the straw is left on the paddies. No rice straw is incorporated **into soil** before the flood period **and that resulted the reduction of CH4 emission**."

2) How to prove that the flood in the previous study has insufficient DO, but there was adequate for this study?

The difference of DO is clear because the location of rice straws is different. We emphasized this by adding the reference of Wassmann et al. in the above paragraph.

3) This recommendation might not be useful and need to open for multidisciplinary discussions.

We revised the manuscript as follows.

"Our results indicate that the main cause of the increase in methane emissions was the incorporation of rice straw into the soil. In contrast, decomposing rice straw in paddy surface-water generated less methane. Thus, decomposing rice straw in paddy surface-water is an effective method to reduce methane emissions **in this area. However, environmental sustainability should be considered the conversion of CH4 to other carbon forms could also result in additional ecological problems. In addition, socioeconomics should be considered since minimizing cropping cycles may not be socially acceptable.**"

The conclusion also added the following sentence.

" **However, the effects of CH4 conversion to other carbon forms and of socioeconomics should be clarified.** "

# Response

**Published:** 2021-01-26

**Authors:** Masato Oda

**Affiliations:** Japan International Research Center for Agricultural Sciences (JIRCAS), Japan

First, the purpose of this study is not to propose a technology. Therefore, in the revised manuscript, we limited our conclusions to the results of the analysis.

 Second, you have pointed out the contradiction with existing studies, but the two are not contradictory.

 The point is not whether there is rice straw in the rice paddies, but whether the straw is incorporating into the soil. In the revised manuscript, I clearly stated: "incorporating into the soil" to avoid misunderstanding.

 The data show that the methane production is obviously different depending on whether the rice straw is in the soil or in the water. Also, if the rice straw is not continuously incorporated into the soil, the amount of methane produced is smaller.

 Methane is produced in a reduced state. In other words, the reduction of methane production in the presence of large amounts of rice straw is evidence of the presence of dissolved oxygen. The revised manuscript explained in detail the continuous supply of dissolved oxygen in the water.

# Peer review: Reviewer response for version 4

**DOI:** 10.5256/f1000research.25143.r61259

**Published:** 2020-03-26

**Authors:** Arika Bridhikitti

**Affiliations:** 1 Environmental Engineering and Disaster Management Program, Mahidol University Kanchanaburi Campus, Kanchanaburi, Thailand

Consistently, the authors have not well communicated in their writing and poorly reviewing the previous studies resulting in devaluing the work despite interesting methodology. For example, the authors cited their own articles, and readers cannot clearly distinguish ones from the previous studies and one of this study. Though, there is some inconsistency between the previous study of Oda and Chiem (2019) — “the absence of rice plants doubles the methane emission from the field because of the lack of methanogenesis inhibition by rice plants”— and the Figure 1 of this study. The authors are not attempting to discuss this inconsistency but try to discuss their new findings without citing any supporting evidence from other previous works.

Is the work clearly and accurately presented and does it cite the current literature?

Yes

If applicable, is the statistical analysis and its interpretation appropriate?

Not applicable

Are all the source data underlying the results available to ensure full reproducibility?

No

Is the study design appropriate and is the work technically sound?

Yes

Are the conclusions drawn adequately supported by the results?

Partly

Are sufficient details of methods and analysis provided to allow replication by others?

No

Reviewer Expertise:

NA

I confirm that I have read this submission and believe that I have an appropriate level of expertise to state that I do not consider it to be of an acceptable scientific standard, for reasons outlined above.

# Response

**Published:** 2020-04-22

**Authors:** Masato Oda

**Affiliations:** Japan International Research Center for Agricultural Sciences (JIRCAS), Japan

Thank you for reviewing and we are sorry to late to respond.

**1) For example, the authors cited their own articles, and readers cannot clearly distinguish ones from the previous studies and one of this study.**

Oda & Chiem (2019) is a field experiment conducted in the year 2018; instead, this work is "a specific analysis of a part of the data set; Introduction L. 15" of an old project (2011-2016). We are sorry that the relation is a little complicated; however, that is a fact. What we call "data analysis" can do at any time.

We welcome if you give us a piece of advice for the way to dissolve this problem.

**2) There is some inconsistency between the previous study of Oda and Chiem (2019) — “the absence of rice plants doubles the methane emission from the field because of the lack of methanogenesis inhibition by rice plants” — and the Figure 1 of this study.**

**The authors are not attempting to discuss this inconsistency but try to discuss their new findings without citing any supporting evidence from other previous works.**

The sentence is in the result section and the inconsistency is discussed in the discussion sections as follows. 

"This can be explained by the accumulation of rice residue from the preceding crops, especially by the rice straw incorporated into the soil, because the contribution of the present rice-derived carbon is small; Discussion L. 2"

Remember that "Just after the 3rd crop, the natural flood starts so the straw is left on the paddies; Introduction L. 10". No rice straw is incorporated before the flood period. No rice straw is incorporated before the flood period. That is the difference in the conditions of Oda & Chiem 2019.

We are waiting that your response.

# Peer review: Reviewer response for version 4

**DOI:** 10.5256/f1000research.25143.r61258

**Published:** 2020-03-17

**Authors:** Kazuyuki Yagi

**Affiliations:** 1 The Joint Graduate School of Energy and Environment (JGSEE), Center of Excellence on Energy Technology and Environment, King Mongkut’s University of Technology Thonburi, Bangkok, Thailand

I read the revised manuscript (ver. 4) and the Author Response (dated on 21 Feb 2020) to my comments on its ver. 2 (12 Nov 2019) that was reported on 19 Feb 2020. As a result, I found that the response was quite unsatisfactory to accept.

 The authors responded to many of my comments that they think the matter of writing style is not constructive. However, I do not agree with that because those comments are not the matter of writing style but that of fundamental requirements for a scientific paper. I cannot understand their statement that ‘because that is largely affected by the background of the main journal we have read’.

 Also, the term ‘natural flood’ or ‘annual flood’ is still unclear. It is written that ‘natural flood of two months (starting from around late September to late October) limits the rice cultivation period. The 1st crop (winter-spring) begins after the natural flood, … (Introduction, 2 ^nd^ paragraph lines 1–4)’. Then, when was the period ‘Flood’ in Figure 1? It is difficult to understand the periods written in the ‘Emission level’ section of Results: ‘after the natural flood (line 11)’, ‘during the flood period and the 1st crop (lines 12–13)’, and ‘the flood period (line 14)’.

 In addition, the response to other minor comments are not satisfactory as listed below:

  

- Management of rice straw: Authors added the average rice straw dry weight, but it is still unclear how much of the straw was returned to the soil.
- Statistical analysis: Authors added 95% CI and the numbers of replication, but not analyzed the significance of difference between seasons.
- After Vo *et al.*, 2018 has published, it cannot say ‘these emissions are not well studied’. Also, the knowledge in the IPCC Guidelines should be cited and included.
- The details of the soil properties should be provided if they have not been published in another paper.
- The reason why the result was not consistent to the ‘previous’ report (Results, Emission level, Lines 13–15) is still unclear. The ‘previous’ report (Oda & Chiem, 2019) reported that rice suppressed overall methane emissions.

 

 In conclusion, because of the result of above-mentioned assessment, I have no other choice to recommend ‘reject’ for this paper, despite that it includes original and remarkable findings that are valuable to the scientific communities.

Is the work clearly and accurately presented and does it cite the current literature?

No

If applicable, is the statistical analysis and its interpretation appropriate?

No

Are all the source data underlying the results available to ensure full reproducibility?

Partly

Is the study design appropriate and is the work technically sound?

Yes

Are the conclusions drawn adequately supported by the results?

Partly

Are sufficient details of methods and analysis provided to allow replication by others?

No

Reviewer Expertise:

biogeochemistry, soil science

I confirm that I have read this submission and believe that I have an appropriate level of expertise to state that I do not consider it to be of an acceptable scientific standard, for reasons outlined above.

# Response

**Published:** 2020-03-23

**Authors:** Masato Oda

**Affiliations:** Japan International Research Center for Agricultural Sciences (JIRCAS), Japan

*The authors responded to many of my comments that they think the matter of writing style is not constructive.

That’s a misunderstanding. That is because we think most of your comments are not the matter of writing style but that of fundamental requirements for a scientific paper. 

*When was the period ‘Flood’ in Figure 1?

You can see the period in Figure 2.

*How much of the straw was returned?

We returned all; therefore, we don't mention how much.

*Significance test

That means significant at 5% level when the 95%CI do not overlap.

*Soil properties

We can show a paper which conducted the same place. The paper shows that the soil is the silty-clay Fluvisol soils (clay 52%, silt 48%, sand 0.3%; FAO Soil Classification; Arai et al. 2015).

*The reason why the result was not consistent to the ‘previous’ report

That is explained in the discussion section as follows. "The reset of emission levels after the annual flood means that the rice straw is decomposed without methanogenesis in water". This high emission resulted from rice straw incorporation.

# Peer review: Reviewer response for version 3

**DOI:** 10.5256/f1000research.24893.r60555

**Published:** 2020-03-04

**Authors:** Arika Bridhikitti

**Affiliations:** 1 Environmental Engineering and Disaster Management Program, Mahidol University Kanchanaburi Campus, Kanchanaburi, Thailand

This improvement has been more scientific sounds. The authors discuss based on the experimental results. The manuscript; however, has major concerns on the unclear conclusion, miscommunication, and writing problems.

**Methods:**

- Rice variety should be firstly introduced in scientific names.

**Results:**

- Unbalance parenthesis when introducing the methane emissions from 1st, 2nd, and 3rd cropping periods.
- The sentence on “The total emission should be higher than that of the 3rd crop: because the absence of rice plants doubles the methane emission from the field (Oda and Chiem, 2019)” is not understandable. What is the total emission? Why does the absence of rice plants cause double emission?
- The sentence on ”In the present study, the pattern was the same as the previous study (Oda and Chiem, 2019)” is also hard to understand. What is the present study? Oda and Chiem (2019) or this study? If the author means this study, it was also not the same as the previous study since no emission peaked at the heading stage.
- Figure 2 should provide additional information on the heading stage, irrigation period, and period of the full canopy development

​​​​​​ **Discussion:**

- the sentence on “the rice straw is decomposed without methanogenesis in the water because the water includes dissolved oxygen” is doubtful. The author should discuss more how CH _4_ forms without methanogenesis and how to be confident that the DO in the water did not deplete for the entire 2-month flooding.

Is the work clearly and accurately presented and does it cite the current literature?

Yes

If applicable, is the statistical analysis and its interpretation appropriate?

Not applicable

Are all the source data underlying the results available to ensure full reproducibility?

No

Is the study design appropriate and is the work technically sound?

Yes

Are the conclusions drawn adequately supported by the results?

Partly

Are sufficient details of methods and analysis provided to allow replication by others?

No

Reviewer Expertise:

Environmental Engineer

I confirm that I have read this submission and believe that I have an appropriate level of expertise to confirm that it is of an acceptable scientific standard, however I have significant reservations, as outlined above.

# Response

**Published:** 2020-03-09

**Authors:** Masato Oda

**Affiliations:** Japan International Research Center for Agricultural Sciences (JIRCAS), Japan

We deeply thank you for giving valuable suggestions.

**Rice variety should be firstly introduced in scientific names.**

 We added.

**Unbalance parenthesis.**

 We corrected.

**The sentence on “The total emission should be higher than that of the 3rd crop: because the absence of rice plants doubles the methane emission from the field (Oda and Chiem, 2019)” is not understandable. What is the total emission? Why does the absence of rice plants cause double emission?**

 We added the value of the total emission and clarified the sentence and added the reason.

**The sentence on ”In the present study, the pattern was the same as the previous study (Oda and Chiem, 2019)” is also hard to understand. What is the present study? Oda and Chiem (2019) or this study? If the author means this study, it was also not the same as the previous study since no emission peaked at the heading stage.**

 We clarified the sentence.

**Figure 2 should provide additional information on the heading stage, irrigation period, and period of the full canopy development.**

 We added the information for the heading stage and the irrigation period. Unfortunately, we don't have data on the period of full canopy development.

 ​​​​​​ **Discussion:**

**the sentence on “the rice straw is decomposed without methanogenesis in the water because the water includes dissolved oxygen” is doubtful. The author should discuss more how CH4 forms without methanogenesis and how to be confident that the DO in the water did not deplete for the entire 2-month flooding.**

 1) We added the explanation for CH4 forms without methanogenesis.

 2) We added a possible explanation for the DO in the water did not deplete.

# Peer review: Reviewer response for version 2

**DOI:** 10.5256/f1000research.23378.r59810

**Published:** 2020-02-19

**Authors:** Kazuyuki Yagi

**Affiliations:** 1 The Joint Graduate School of Energy and Environment (JGSEE), Center of Excellence on Energy Technology and Environment, King Mongkut’s University of Technology Thonburi, Bangkok, Thailand

The study reports original and remarkable findings in increasing methane emissions from triple cropping of rice. Although it is not clearly stated in the manuscript, there are very few studies that reported methane emissions from triple rice cropping. In addition, the major finding of this study, that is the increasing methane emissions in the 2nd crop relative to the 1st one, and further in the 3rd crop, in response to the accumulation of rice residue from the preceding crops, is a remarkable fact for understanding the intensity of emissions, as well as designing options for climate change mitigation. Therefore, the research is recognized to be valuable in reporting in an international scientific journal.

 However, at the same time, I have to report that current manuscript has substantial lack of basic components and requirements as a scientific paper at each section throughout the manuscript, even though this paper has submitted as a Brief Report. Note that filling those lack of details is essential as a scientific paper to make sure that readers have enough information to understand the description of the work. The specific points of problems are listed below. As a result, I can approve the manuscript only after it was revised by responding to my comments in an appropriate manner.

**Introduction: **

- Background (the need for this study) and objectives of the study should be clearly presented. Why did you study methane emissions from paddy fields? What gaps of knowledge did you try to approach? What are the objectives of this study?

 

**Methods:**

- The major finding of this study is the importance of straw incorporation into the soil after harvesting rice for increasing methane emissions in the following season. From this viewpoint, it is requested to report management of rice straw after every harvest more quantitatively in detail in the Method section. It is required to provide the amount of rice straw (and preferably that of stubbles and roots) returned to the fields from previous harvest, because it is essential to discuss the increase in methane emissions. Otherwise, it is vague to discuss the emission pattern in Discussion. 

 

**Data analysis: **

- The statistical analysis for judging the differences in methane emissions among the seasons is insufficient, because no statistical analysis was made for the data in Figure 1. As a result, it is difficult to conclude the differences in methane emissions among the seasons. 

 

**Results:**

- In this section, the results of experiment should be simply presented. Information of previous studies and discussion with them should be presented in Introduction or Discussion sections, respectively. From this, the sentences in Line 1–6 of ‘Emission level’ part and in Line 1–11 of ‘Emission pattern’ part should be moved.

 

**Data presentation: **

- The amounts of total seasonal methane emission should be numerically presented, at least those of five-year average for each crop with the values of interannual variation.

 

**Discussion: **

- There is fundamental lack of discussion with referring to previous studies by other researches who reported methane emissions from paddy fields. A sweeping revision is necessary in this section.

 

 In addition, some minor comments are listed below:

1. Throughout the manuscript, the term ‘natural flood’ or ‘annual flood’ should be corrected to ‘fallow flood’ because it is confusing. ‘After natural (annual) flood’ can be ‘during the fallow flood period after harvesting the 3rd crop’.
2. Abstract, Line 2: The statement that ‘these emissions are not well studied’ is incorrect. A number of field measurements for methane emissions from paddy fields in the Mekong Delta have been published, such as Vo *et al.*, 2018 (already cited in this paper), and references cited in the paper.
3. Introduction, 1st paragraph, Line 9: The statement that ‘this has not been well studied’ is incorrect. IPCC Guidelines provides the quantitative effects of rice straw incorporation on increasing methane emissions as a scaling factor based on a number of field measurements including those in the Mekong Delta. 
4. Introduction, 3rd paragraph, Lines 6–8: The reference that shows effectiveness of the strategy should be cited.
5. Methods, Site description: It is suggested to report the type of soil and its characteristics, because the information is essential for discussing the intensity of methane emissions from paddy fields and is not presented in the previous paper. I would suggest to report, at least, soil texture, organic carbon content, and pH, all of which are recognizes as the major factors controlling methane emissions. 
6. Figures 1 and 2: The period of data ‘Flood’ is confusing. It is recommended to correct it with ‘Fallow flood’.
7. Results, Emission level, Lines 9–11: The emission level doubled in the 2nd crop, and tripled in the 3rd crop, compared with that in the 1st crop, then reset after the fallow flood. Same correction should be made at Discussion, Emission pattern, Lines 1–3.
8. Results, Emission level, Lines 13–: The total emission during the fallow flood should be higher …
9. Results, Emission level, Lines 13–15: Why the result was not consistent to the ‘previous’ report. Please discuss it.
10. Results, Emission pattern, Lines 1–11: This statement is not the results in this study. Therefore, it should be moved to Discussion. Also, the difference of data in ‘previous’ study and this one should be clarified. Understanding from the ‘previous’ paper (Oda & Chiem, 2019), the ‘previous’ study reported the results of experiments during 2016 and 2017, whereas this study during 2011 and 2016. If it is correct, using the term ‘previous’ is not appropriate.
11. Discussion, 2nd paragraph, Line 6: no methane emission -> little methane emission

Is the work clearly and accurately presented and does it cite the current literature?

No

If applicable, is the statistical analysis and its interpretation appropriate?

No

Are all the source data underlying the results available to ensure full reproducibility?

Partly

Is the study design appropriate and is the work technically sound?

Yes

Are the conclusions drawn adequately supported by the results?

Partly

Are sufficient details of methods and analysis provided to allow replication by others?

No

Reviewer Expertise:

biogeochemistry, soil sciences

I confirm that I have read this submission and believe that I have an appropriate level of expertise to confirm that it is of an acceptable scientific standard, however I have significant reservations, as outlined above.

# Response

**Published:** 2020-02-21

**Authors:** Masato Oda

**Affiliations:** Japan International Research Center for Agricultural Sciences (JIRCAS), Japan

Thank you for deeply considering our manuscript. We considering your helpful comments and improved the manuscript. However, we are afraid that adopting the term "fallow flood" is not right in case of the Mekong Delta triple cropping rice. In addition, we think the matter of writing style is not constructive because that is largely affected by the background of the main journal we have read. Finally, we are glad to have helpful comments from you. The details of our response are as follows.

 Introduction:

 •    Background (the need for this study) and objectives of the study should be clearly presented. Why did you study methane emissions from paddy fields? What gaps of knowledge did you try to approach? What are the objectives of this study?

 *As you mention "there are very few studies that reported methane emissions from triple rice cropping." That is the reason. We described more clearly the aim of the study.

 Methods:

 •    The amount of rice straw 

 *They were added.

 Data analysis:

 •    No statistical analysis was made for the data in Figure 1.

 *You mention the significant tests, don’t you? Now, that is no longer recommended; instead, 95% confidential interval is recommended (https://www.nature.com/articles/d41586-019-00857-9). That can be known from the SD and the n. However, we found that the figure is needed correction of the way of calculation of the mean (each crop has three replications but means should be calculated by year, and the n should be changed from 15 to 5). In addition, the error bars were not SD but were SE. We corrected the figure and show the 95% confidential interval (CI). We deeply thank that you give us a chance to correct the error bars.

 Results:

 •    In this section, the results of experiment should be simply presented. Information of previous studies and discussion with them should be presented in Introduction or Discussion sections, respectively. From this, the sentences in Line 1–6 of ‘Emission level’ part and in Line 1–11 of ‘Emission pattern’ part should be moved.

 *This is a matter of writing style. The consideration of the values using citations is common in the results section.

 Data presentation:

 •    The amounts of total seasonal methane emission should be numerically presented, at least those of five-year average for each crop with the values of interannual variation.

 *We showed them as a range, but we changed that as each average.

 Discussion:

 •    There is fundamental lack of discussion with referring to previous studies by other researches who reported methane emissions from paddy fields. A sweeping revision is necessary in this section.

 *This is a matter of writing style. We cited the studies in the introduction section. As you know, they are few.

 In addition, some minor comments are listed below:

 1.    Throughout the manuscript, the term ‘natural flood’ or ‘annual flood’ should be corrected to ‘fallow flood’ because it is confusing. ‘After natural (annual) flood’ can be ‘during the fallow flood period after harvesting the 3rd crop’.

 *There is no fallow flood. We show the condition by describing the cropping days and the intervals in the manuscript.

 2.    Abstract, Line 2: The statement that ‘these emissions are not well studied’ is incorrect. A number of field measurements for methane emissions from paddy fields in the Mekong Delta have been published, such as Vo et al., 2018 (already cited in this paper), and references cited in the paper.

 *We cited the description “not well studied” of Vo et al. The situation has not changed after a year.

 3.    Introduction, 1st paragraph, Line 9: The statement that ‘this has not been well studied’ is incorrect. IPCC Guidelines provides the quantitative effects of rice straw incorporation on increasing methane emissions as a scaling factor based on a number of field measurements including those in the Mekong Delta. 

 *See above. We think this kind of rebuttal is not constructive.

 4.    Introduction, 3rd paragraph, Lines 6–8: The reference that shows effectiveness of the strategy should be cited.

 *This is a matter of writing style. Showing the main finding of the work at the end of the introduction section is common.

 5.    Methods, Site description: It is suggested to report the type of soil and its characteristics, because the information is essential for discussing the intensity of methane emissions from paddy fields and is not presented in the previous paper. I would suggest to report, at least, soil texture, organic carbon content, and pH, all of which are recognizes as the major factors controlling methane emissions. 

 *The details of the soil properties will be published in another paper. That is the reason why we don’t describe the details. We think this kind of problem that comes from closed science should be diminished in the future.

 6.    Figures 1 and 2: The period of data ‘Flood’ is confusing. It is recommended to correct it with ‘Fallow flood’.

 *We think the word “Fallow flood” imagines a fallow paddy with ponding water. The paddy in the Mekong Delta is really a flood, a natural disaster.

 7.    Results, Emission level, Lines 9–11: The emission level doubled in the 2nd crop, and tripled in the 3rd crop, compared with that in the 1st crop, then reset after the fallow flood. Same correction should be made at Discussion, Emission pattern, Lines 1–3.

 *You understood correctly without the phrase, “compared with that in the 1st crop”. Flood is not fallow. We think this kind of rebuttal is not constructive.

 8.    Results, Emission level, Lines 13–: The total emission during the fallow flood should be higher …

 *We cannot agree to use the term.

 9.    Results, Emission level, Lines 13–15: Why the result was not consistent to the ‘previous’ report. Please discuss it.

 *That is in the discussions section. The reset of emission levels after the annual flood means that the rice straw is decomposed without methanogenesis in water because the water includes dissolved oxygen.

 10.    Results, Emission pattern, Lines 1–11: This statement is not the results in this study. Therefore, it should be moved to Discussion. Also, the difference of data in ‘previous’ study and this one should be clarified.

 *Generally, “what the data are” is describes in the results section; instead “what the data meaning” is describes in the discussions sections. According to these criteria, Lines 1-11 is suitable for the results section. 

 Understanding from the ‘previous’ paper (Oda & Chiem, 2019), the ‘previous’ study reported the results of experiments during 2016 and 2017, whereas this study during 2011 and 2016. If it is correct, using the term ‘previous’ is not appropriate.

 *Only published studies can cite in the paper. They are called previous studies, not future studies. We think this kind of rebuttal is not constructive.

 11.    Discussion, 2nd paragraph, Line 6: no methane emission -> little methane emission

 *Theoretically and practically "no" but we follow your recommendation for avoiding unconstructive rebuttal. Thank you.

# Peer review: Reviewer response for version 2

**DOI:** 10.5256/f1000research.23378.r56459

**Published:** 2020-02-10

**Authors:** Arika Bridhikitti

**Affiliations:** 1 Environmental Engineering and Disaster Management Program, Mahidol University Kanchanaburi Campus, Kanchanaburi, Thailand

I still have concerns about the accuracy of the methodology applied for this work since the result is double the maximum value given by prior studies conducted in the same region. Though the author is convinced that extremely high CH _4_ is usual in the studied areas but no scientific evidence is shown in context.

 Furthermore, the suggestion given by the authors on the decomposing rice straw in flooding field may not be feasible due to a lower gain of the farmers. The authors are convinced that the yield in the tripled crop is low, and the farmers will have much more profit by practising organic farming. This discussion is out of the ring because there is no evidence on rice yield, and organic farming is out of the scope of this work.

Is the work clearly and accurately presented and does it cite the current literature?

Yes

If applicable, is the statistical analysis and its interpretation appropriate?

Not applicable

Are all the source data underlying the results available to ensure full reproducibility?

No

Is the study design appropriate and is the work technically sound?

Yes

Are the conclusions drawn adequately supported by the results?

Partly

Are sufficient details of methods and analysis provided to allow replication by others?

No

Reviewer Expertise:

Environmental Engineer

I confirm that I have read this submission and believe that I have an appropriate level of expertise to state that I do not consider it to be of an acceptable scientific standard, for reasons outlined above.

# Response

**Published:** 2020-02-12

**Authors:** Masato Oda

**Affiliations:** Japan International Research Center for Agricultural Sciences (JIRCAS), Japan

**1) Methodology**

We followed the standard method so replication by others is capable. As you know,  to say overestimate requires grounds; however, data for methane emission in triple cropping rice is little. Note conditions of the reference we indicate are milder than that of the present study. We think the discussion should start waiting for more data.

Importantly, the theme of this brief report is not the emission levels but is the pattern, especially the role of the flood period reducing methane emission. They are not affected by whether we overestimated or underestimated. 

**2) Discussions**

We guess that you might be confusing the personal response and the manuscript because of the system. That is actually "out of the ring".

We hope you agree with the above explanations.

# Response

**Published:** 2020-02-26

**Authors:** Masato Oda

**Affiliations:** Japan International Research Center for Agricultural Sciences (JIRCAS), Japan

We found an example of high emission in Japan ( https://www.naro.affrc.go.jp/org/warc/research_results/skk_seika/h06/94017-z1.jpg). The total amount roughly estimated about 2000 kg CH4 ha-1 crop-1. The result obtained under the condition of 5 Mg of wheat straw and 6 Mg of rice straw. Wheat straw is rapidly decomposed than rice straw. The fresh rice straw in the Mekon Delta triple-cropping rice is considered the middle of those organic materials. We added the information of the quantity of applied rice straw to the revised manuscript. That is about 9 Mg per crop. We think this additional information provides enough reason to understand the high emission in our results.

# Peer review: Reviewer response for version 2

**DOI:** 10.5256/f1000research.23378.r56458

**Published:** 2019-11-13

**Authors:** Tran Dang Hoa

**Affiliations:** 1 University of Agriculture and Forestry, Huế University, Hue, Vietnam

The revised version is good enough. I agree with the responses of the authors.

Is the work clearly and accurately presented and does it cite the current literature?

Yes

If applicable, is the statistical analysis and its interpretation appropriate?

Partly

Are all the source data underlying the results available to ensure full reproducibility?

Yes

Is the study design appropriate and is the work technically sound?

Yes

Are the conclusions drawn adequately supported by the results?

Partly

Are sufficient details of methods and analysis provided to allow replication by others?

Partly

Reviewer Expertise:

Low carbon rice production

I confirm that I have read this submission and believe that I have an appropriate level of expertise to confirm that it is of an acceptable scientific standard.

# Peer review: Reviewer response for version 1

**DOI:** 10.5256/f1000research.22010.r54181

**Published:** 2019-10-23

**Authors:** Tran Dang Hoa

**Affiliations:** 1 University of Agriculture and Forestry, Huế University, Hue, Vietnam

The measurement of GHG emission from paddy fields in Mekong Delta have been carried out by several authors. However, understanding of GHG emission from paddy field by triple seasons isn't well known. This study contributed some good information on GHG emission, focusing on CH _4_ to understanding how to reduce GHG emission from the paddy field.

 My commends are below:

**Introduction:**

- Introduction should be given a general information on rice production in Mekong Delta in order to explain this study is a representative.

**Method:**

- Site description should be more clear, such as, what about the field practices of the farmer on their fields? Were the three observed fields similar in these practices?  What about the water management in the fields? The author said the fields was continuously flooding, but how about water level/regime in three observed fields? How about straw management? What about the height of stubble for each season? How was about fertilization?...
- Methane measurement: The author said the closed chamber method described by Oda &Chien, 2019. However, a brief description should be added. What time of the day were GAS samples take? How was CH _4 _calculated?
- Statistical analysis: A one-way ANOVA should be analysed to compare the means among season?

**Discussion:**

- The authors just explained the difference of CH _4_ emission among seasons was due to straw incorporation into the soil. However, I did not see any information on straw incorporation in the observed fields (as above commented). Other factors such as rice varieties, weather, fertilization... should be discussed. In this case, these factors differed among seasons, why did only straw incorporation influence on CH _4_ emissions?

**Conclusion: **

- As above commented, the conclusion on CH _^4^_ emission increase being due to straw incorporation is not clear. 

Is the work clearly and accurately presented and does it cite the current literature?

Yes

If applicable, is the statistical analysis and its interpretation appropriate?

Partly

Are all the source data underlying the results available to ensure full reproducibility?

Yes

Is the study design appropriate and is the work technically sound?

Yes

Are the conclusions drawn adequately supported by the results?

Partly

Are sufficient details of methods and analysis provided to allow replication by others?

Partly

Reviewer Expertise:

Low carbon rice production

I confirm that I have read this submission and believe that I have an appropriate level of expertise to state that I do not consider it to be of an acceptable scientific standard, for reasons outlined above.

# Response

**Published:** 2019-11-05

**Authors:** Masato Oda

**Affiliations:** Japan International Research Center for Agricultural Sciences (JIRCAS), Japan

Thank you very much for giving valuable comments.

1. Introduction: a general information in order to explain this study is a representative

Thank you. We added.

2. Methods

2-1. Site description:

The sentence was improved to clarify that the present study was an observation of typical farmer’s paddies mentioned in the introduction (including rice straw treatment).

2-2. Farmers practices:

Thank you. We added the reference. The height of the stubble is about 30 cm. The daily average water levels were monitored with water level loggers at the corner of the fields; the average levels were 2.0 cm (−0.6 to 6.1 cm) until drained (the data will be published in another paper).

2-3. Methane measurement:

Thank you. We added.

3. Statistical analysis:

Figure 1 is clear enough to omit ANOVA (see the standard deviation (not SE)).

4. Discussion:

The information on straw incorporation in the observed fields is mentioned in the introduction. Rice straw incorporation enhances methane emission. We added a referred to our previous study that is a kind of short review of the effect of straw incorporation.

# Peer review: Reviewer response for version 1

**DOI:** 10.5256/f1000research.22010.r54183

**Published:** 2019-10-18

**Authors:** Arika Bridhikitti

**Affiliations:** 1 Environmental Engineering and Disaster Management Program, Mahidol University Kanchanaburi Campus, Kanchanaburi, Thailand

The idea of estimating methane emissions from rice paddy fields is not novel. Many publications, including those conducted in SE Asia, have been widely accepted, such that collected and reported in the 1996 IPCC Guidelines for National Greenhouse Gas Inventories: Reference Manual. The study on triple rice cropping in the Mekong River Delta is quite interesting since few studies carried on such the cropping system, but usually estimating the total flux based upon one single cropping experiment.

 My first significant concern in this work is involved with the methodology of methane sampling and

 analysis. The closed chamber method used in this study (Oda and Chiem, 2019) had not yet been validated with other traditional closed chamber studies. Furthermore, methane emission using this

 method seems to return significantly higher methane emissions compared to those emissions

 reported worldwide in the IPCC report. Since very much high emission rates can result in

 significantly overestimating methane emissions from the SE Asia region, I suggest the authors

 carefully discuss the reliability of the proposed method in comparison to the traditional method.

 My last concern is on the suggestions given by the authors. They claim that decomposing rice straw in paddy surface-water (nonrice period) is an effective method to reduce methane emissions. This

 suggestion is not conforming to the fact that the wetland system is a significant source of methane

 emission. I think the author could mean lower methane emission during the nonrice flooding

 period, but the conclusion written in the manuscript is not clear that way. Although, I think the

 conclusion given by the authors is not feasible since promoting long-term nonrice flooding instead

 of rice cropping could mean lower rice yield.

Is the work clearly and accurately presented and does it cite the current literature?

Yes

If applicable, is the statistical analysis and its interpretation appropriate?

Not applicable

Are all the source data underlying the results available to ensure full reproducibility?

No

Is the study design appropriate and is the work technically sound?

Yes

Are the conclusions drawn adequately supported by the results?

Partly

Are sufficient details of methods and analysis provided to allow replication by others?

No

Reviewer Expertise:

NA

I confirm that I have read this submission and believe that I have an appropriate level of expertise to confirm that it is of an acceptable scientific standard, however I have significant reservations, as outlined above.

# Response

**Published:** 2019-11-05

**Authors:** Masato Oda

**Affiliations:** Japan International Research Center for Agricultural Sciences (JIRCAS), Japan

Thank you for giving valuable comments for improving our manuscript.

1. The methodology of methane sampling and analysis.

Our methodology is the traditional method and there are high emission data in An Giang province. The water management conditions are better than our site because of the full-dike system. Thus, we think our data are reliable. We added the explanations.

2. Suggestion by authors

We agree with you that the wetland system is a significant source of methane emission. We also agree with you that the methane emission in the paddy field is extremely high compared to the simple wetland. Our finding is that the cause of the high emission in the paddy field is the incorporation of rice straws. The recent spread of combine harvesting decreases the need for rice straw burning. The methane emission will increase much more. Therefore, decreasing the methane emission of the paddy field to the levels of the simple wetland is still significant according to the data. Our suggestion is directly led by the results of the present study; although there are many other indirect options.

For the yield of rice, we think by the profit. A recent study reported that the profit of triple rice is only 6% higher than double cropping ( J Environ Manage. 2018). However, utilizing rice straw enables organic farming and that brings much profit by the high unit price. We are trying to establish cultivation practice. Furthermore, using the ratooning triple cropping is also possible.
